# Using ERG inquiry to evoluate otoplasty satisfaction in an otorhinolaryngology medical residency training hospital

**DOI:** 10.1590/S1808-86942012000100018

**Published:** 2015-10-20

**Authors:** Silvio Antonio Monteiro Marone, Tarcisio Aguiar Linhares Filho, Renato Tadao Ishie, Otavio Borio Dode, Bernardo Campos Faria, Jose Luiz Teixeira Rodrigues, Marcio Antonio De Souza

**Affiliations:** a Doctoral degree in otorhinolaryngology, Medical School, Sao Paulo University–FMUSP (Full professor of otorhinolaryngology, Medical School, Campinas Pontifical University. Director of the medical residency program in otorhinolaryngology, Santa Marcelina Hospital); b Medical resident in otorhinolaryngology, Santa Marcelina Hospital (3^rd^ year); c Otorhinolaryngologist, ABORL.; d Faculty member of the Otorhinolaryngology Discipline, Atenas School, Paracatu, MG (Otorhinolaryngologist); e Otorhinolaryngologist, ABORL. Tutor of facial esthetic surgery, Santa Marcelina Hospital. Santa Marcelina Hospital.

**Keywords:** ear auricle, patient satisfaction, surgery, plastic

## Abstract

The ear deformity surgery intervention impact on psychological and self-esteem aspects, in adults and children, is well documented. Recently, the studys are focused on patient satisfaction, funcional result and impact on quality of life. Any modification on patient's quality of life has been a challenge. The use of valid and established questionnairies, like Glasgow Benefit Inventory (GBI), assists on data analyse, turning it consistent.

**Aim:**

The aim of this study is to evaluate the impact on patients quality of life after otoplasty, through the GBI questionnaire.

**Methods:**

Retrospective study including patients underwent otoplasty, within july of 2009 to july of 2010. The data were collected through questionnaire applied by medical resident on 90 post-surgical return.

**Results:**

36 patients answered the questionnaire. There was increase on patients quality of life demonstrated by positive mediana obtained through out questinnaire. There were no significantly differences between age and sex.

**Conclusion:**

The patients are satisfied with post-surgical results. There was increase on patients quality of life conform positive results obtained. The use of GBI showed easy and elucidative.

## INTRODUCTION

A protruding ear is the most common congenital anomaly of the outer ear. It affects about 5% of the general population; its transmission is autosomal dominant. Although the biological consequences are benign, several studies have demonstrated the psychological suffering, emotional trauma, and behavioral changes that result from this deformity, especially among children^1^. This facial anomaly may give rise to low self-esteem, anxiety, behavioral problems, and social withdrawal^2^. In particular, protruding ears may evoke ridicule from others and result in significant emotional disorders^3^, which reduces the health-related quality of life (HRQoL). It is currently clear that a decreased HRQoL is associated with impaired daily activities such as school and work^4^, ^5^.

Several studies have documented the importance of surgery for correcting ear deformities and other craniofacial abnormalities in reducing the psychological pain and increasing the self-esteem of adult and child patients^6^, ^7^, ^8^. Otoplasty techniques are easy to learn and very useful for training medical residents^9^. The main goal of therapy is to attain a symmetrical, well-formed, and acceptable position of the ear, which will contribute towards the satisfaction of patients and family members.

Recent studies have focused on the results of treatment that focus on patients by noting their satisfaction, functional result, and impact on the quality of life.

Quantifying changes in the HRQoL has been a challenge. The use of valid and well-studied inquiries, such as the Glasgow Benefit Inventory (GBI), helps gather data and add to their consistency^10^.

There is a relative paucity of data in the literature on the impact on the quality of life of patients undergoing otoplasty, especially in the context of medical resident training. Studies assessing the HRQoL may help guide how esthetic procedures are done to attain more therapeutic efficacy and to underline how important it is to apply this procedure in medical training programs.

The purpose of this study was to assess the satisfaction of patients undergoing otoplasty carried out by medical residents at our institution, based on the GBI; a second purpose was to assess the functionality of this questionnaire.

## METHODS

The CEP/CSSM approved this project (no. 51/09). It consisted of a retrospective study that enrolled all patients undergoing otoplasty by medical residents supervised by a tutor who is a specialist in otorhinolaryngology, from July 2009 to July 2010.

Patients or their caretakers signed a free informed consent form before taking part in this study; the form was handed in at the interview.

### Series

Participants of both sexes were enrolled based on the criteria below. Inclusion criteria:

- agreeing with and signing the free informed consent form – adult patients or parents or caretakers in the case of children.
- patients aged 12 years or more. Minors were required to be accompanied by parents or caretakers to help them with the questionnaire.

Exclusion criteria:

- not agreeing with and not signing the free informed consent form – adult patients or parents or caretakers in the case of children.
- inability to understand and/or answer the questionnaire; microtia; genetic conditions including the craniofacial syndrome; altered ear lobe only; unilateral deformity. Patients with other diseases (diabetes, uncontrolled arterial hypertension, immune deficiency, abnormal coagulation tests, cardiopathies) that contraindicated surgery, and patients with evident emotional disorders were not operated.

### Method

Data were gathered through a questionnaire that medical residents gave to patients on at least the 90-day postoperative visit. Discharged patients or those lost to follow-up were invited by telephone. Parents or caretakers accompanied minors and helped them with the questionnaire.

Patients underwent otoplasty under local anesthesia (infiltration of 2% lidocaine and vasoconstrictor – 1:40.000 adrenalin). The surgical technique consisted of making an incision posterior to the ear auricle, followed by weakening by abrading the ear cartilage; then modeling the anti-helix and the conchomastoid angle is reduced by suturing with a non-absorbable suture. The excess conch cartilage is removed, if needed. Patients were evaluated on postoperative days 2, 7, 14, 30, 60, and 90.

The HRQoL questionnaire was applied to patients; the GBI was created especially for procedures and interventions in otorhinolaryngology. The version we used had been translated and adapted for the local culture from the English language. Prior studies had shown the GBI to be reproducible, valid, and responsive^10^. The GBI questionnaire has a total score and three sub-scales. Answers associated with each surgical intervention in otorhinolaryngology while elaborating the GBI showed that each subscale contributed to different aspects of benefits to patients^10^. The first subscale assesses general health, where health is defined as a general perception of physical, social, and psychological well-being^11^: questions 1 to 6, 9, 10, 14, and 16 to 18. The second subscale assesses social support, well being in social relationships: questions 7, 11, and 15. The third subscale assesses physical health: questions 8, 12, and 13. The GBI score was developed originally as ranging from −100 to 100; a positive score means increased quality of life secondary to the procedure that was studied.

We added four of our own questions to the questionnaire. The first three assessed the interviewee's satisfaction with the treatment and the fourth question evaluated whether the patient had any difficulty in answering the questionnaire. The questionnaire with 22 questions is annexed to this study (Annex I).

The IBM-SPSS for Windows was used for data analysis. The Mann-Whitney U test and the Wilcoxon test were applied for comparing both groups. Results were expressed as means +/- standard deviation, or median, and 25 and 75 percentiles. A statistically significant difference was *p*<0.05.

## RESULTS

Seventy-six (76) patients underwent otoplasty at our institution from 03/07/2010 to 01/07/2010. Thirty-six (46%) patients answered the questionnaire. Most of the failures were not returning for the minimum follow-up period.

The mean age of patients was 19.28 years (SD ± 8.92, ranging from 10 to 46 years). Gender distribution: 21 females (58%) and 15 males (42%).

**Chart 1:**
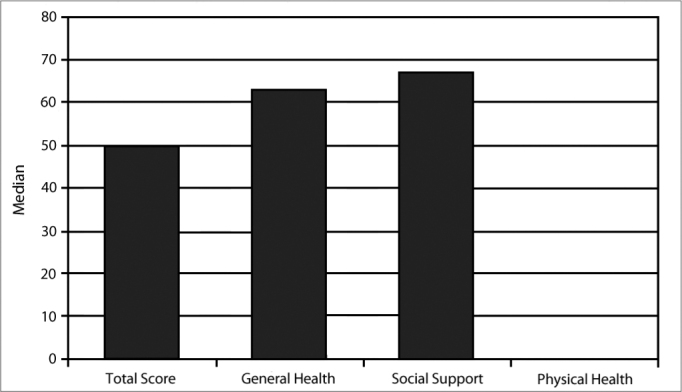
Glasgow Benefit Inventory and its subscales (medians).

The median of the total GBI score was 50 (minimum 0, maximum 100); the value was 33 for the 25^th^ percentile (p-25) and 63 for the 75^th^ percentile (p-75). The median was 63 in the general health subscale (minimum 0, maximum 100); the value was 43 for p-25 and 77 for p-75. The median was 50 (minimum 0, maximum 100) in the social support subscale; the value was 0 for p-25 and 67 for p-75. The median was 0 (minimum −67, maximum 100) in the physical health subscale; the value was 0 for p-25 and 33 for p-75 (Chart 1).

**Chart 2:**
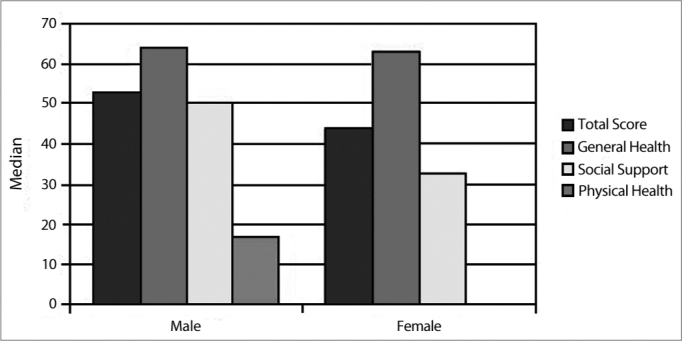
Median values according to gender.

There was no significant gender difference. The median of the total GBI score was 53 (minimum 14, maximum 72) for males; the value was 33 for the 25^th^ percentile (p-25) and 67 for the 75^th^ percentile (p-75). The corresponding median value in females was 44 (minimum 0, maximum 100); the value was 31 for the 25^th^ percentile (p-25) and 58 for the 75^th^ percentile (p-75) (*p*=0.547). The median of the general health subscale in males was 64 (minimum 21, maximum 92); the value was 33 for p-25 and 86 for p-75. The corresponding median value for females was 63 (minimum 0, maximum 100); the value was 48 for p-25 and 76 for p-75 (*p*=0.825). The median of the social support subscale was 50 (minimum 0, maximum 83) in males; the value was 17 for p-25 and 67 for p-75. The corresponding value in females was 33 (minimum 0, maximum 100); the value was 0 for p-25 and 67 for p-75 (*p*=0.309). The median in the physical health subscale was 17 (minimum −17, maximum 67) in males; the value was 0 for p-25 and 33 for p-75. The corresponding value in females was 0 (minimum −67, maximum 100); the value was 0 for p-25 and 17 for p-75 (*p*=0.279). (Table 1; Chart 2).

**Table 1:** Median values according to gender.

Gender		Median	Minimum	Maximum	Percentile 25	Percentile 75
Female	GBI total score	44	0	100	31	58
	General health subscale	63	0	100	48	76
	Social support subscale	33	0	100	0	67
	Physical health subscale	0	-67	100	0	17

Male	GBI total score	53	14	72	33	67
	General health subscale	64	21	92	33	86
	Social support subscale	50	0	83	17	67
	Physical health subscale	17	-17	-67	0	33

**Chart 3:**
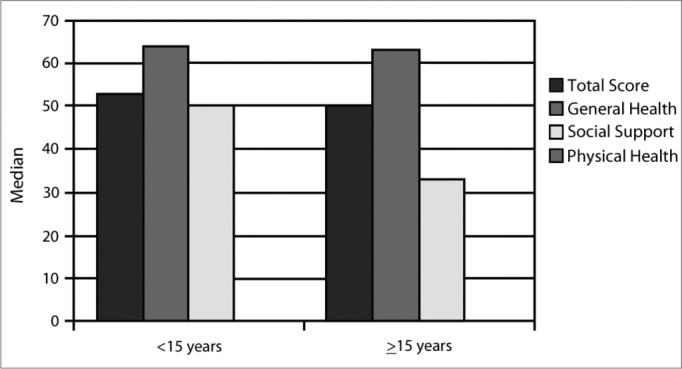
Median values according to age.

There were no differences among results for different age groups. The median of the total GBI score for subjects aged less than 15 years was 53 (minimum 14, maximum 83); the value was 33 for the 25^th^ percentile (p-25) and 64 for the 75^th^ percentile (p-75). The median of the general health subscale was 64 (minimum 21, maximum 100); the value was 36 for p-25 and 82 for p-75. The median of the social support subscale was 50 (minimum 0, maximum 100); the value was 17 for p-25 and 67 for p-75. The median in the physical health subscale was 0 (minimum 0, maximum 67); the value was 0 for p-25 and 33 for p-75. The results for subjects aged 15 years or more were: the median of the total score was 50 (minimum 0, maximum 100); the value was 31 for the 25^th^ percentile (p-25) and 61 for the 75^th^ percentile (p-75) (*p*=0.634). The median of the general health subscale was 63 (minimum 0, maximum 100); the value was 48 for p-25 and 76 for p-75 (*p*=0.704). The median of the social support subscale was 33 (minimum 0, maximum 100); the value was 0 for p-25 and 67 for p-75 (p=0.526). The median of the physical health subscale was 0 (minimum −67, maximum 100); the value was 0 for p-25 and 33 for p-75 (*p*=0.924) (Table 2; Chart 3).

**Table 2:** Median values according to age.

			Median	Minimum	Maximum	Percentile 25	Percentile 75
Age	Less than 15 years	GBI total score	53	14	83	33	64
		General health subscale	64	21	100	36	82
		Social support subscale	50	0	100	17	67
		Physical health subscale	0	0	67	0	33

	15 years or more	GBI total score	50	0	100	31	61
		General health subscale	63	0	100	48	76
		Social support subscale	33	0	100	0	67
Physical health subscale		0	-67	100	0	33

The results of the Yes/No questions were: 35 patients (97%) answered Yes to satisfaction with the esthetic result of surgery; one patient (3%) answered No to this question. Thirty-five patients (97%) answered Yes to referral of our institution to friends/relatives for undergoing the same procedure; one patient (3%) answered No to this question. For the question about undergoing another esthetic procedure at our institution, 32 patients (89%) answered Yes and four patients (11%) answered No. About difficulty in answering the questionnaire, 31 subjects (86%) answered No, and five subjects (14%) answered Yes.

There were six cases (8%) with postoperative complications five had minor complications that were corrected at the time of the diagnosis: a case of an oto-hematoma, two cases of cellulitis of the ear auricle, and two cases with surgical wound infection; these cases progressed without loss of the esthetic result at the end of therapy. There was a case of keloid of the surgical wound, which had not been resolved at the time the inquiry was done. All of these patients answered the questionnaire.

## DISCUSSION

Among all medical specialties, including facial esthetic surgery, there is a growing search for objective data about patient satisfaction and changes in the quality of life as a result of therapy. There is a worldwide search for research methods that objectively assess these parameters. A need for concrete technical, social and economic, and scientific justifications have fostered an interest in satisfaction/quality of life assessments either to adopt novel therapies or to replace or consolidate classical treatments. There is also a need to generate data to support evidence-based medicine.

Otoplasty is a common procedure for correcting protruding ears. Several studies have been published about the complications and results of this procedure, depending on each surgeon's perspective. However, there are few objective evaluations of patient satisfaction with this procedure.

Using the GBI seemed adequate to us. It was designed specifically for otorhinolaryngological procedures and has been validated and well-studied^10^. Furthermore, the moment at which the questionnaire was presented to patients, in a retrospective approach, was more sensitive and closely related with patient satisfaction, as Fischer et al. have demonstrated^11^.

The main difficulty was to have patients return to the scheduled postoperative visits. Many patients in our sample did not return for the visit, and many were lost to follow-up. Patients informally reported factors such as waiting time, the number of patients in waiting rooms, distance from their households to the outpatient unit, difficulties in using public transport, among other reasons.

There was a substantially increase in the total score of the GBI and in the subscales general health and social support after otoplasty. There were no gender differences. Schwentner et al.^7^ demonstrated in their inquiry that the follow-up time did not alter the GBI results; this finding suggests that otoplasty yields a lasting effect on patient satisfaction^12^. Thus, although many patients were lost to follow-up by the third postoperative month, the results can still be considered representative. Analysis of the Yes/No answers corroborates the improvement in quality of life in our sample.

A further point is that the questionnaire is easy to understand; the difficulty also varies according to age. There were no difficulties in filling in the questionnaire, as noted in the answers to question 22 (Did you find it hard to answer this questionnaire?). The results would have varied according to age groups – in the case of older children and adolescents – if there had been variations in understanding the questionnaire; this did not occur (Table 2).

The limitations of this study are the inherent biases that are part of retrospective inquiries, such as the recall bias. The present study is limited to the experience of patients that were operated some time before filling in the questionnaire. Recalling the surgery and its effects may be more difficult after some time, which affects the outcome. Furthermore, the GBI is a measure of patient benefit, rather than health status per se. It does not offer a measure of the patient's health before and after surgery. Finally, a translated and culturally adapted questionnaire may yield different inferences about differing populations, even if the same intention is applied. Validation of the GBI questionnaire for Portuguese would be ideal for comparative inferences.

## CONCLUSION

We believe that otoplasty is an adequate surgical technique for treating protruding ears, even when carried out by medical residents. In this study, patients were satisfied with the postoperative results. Quality of life was increased as shown by the positive results. The GBI appeared to us an easy and elucidative questionnaire.

## ANNEX I The Glasgow Benefit Inventory questionnaire.

1. Have the results of the otoplasty affected your daily activities?
  ( )Much worse ( )A little worse ( )No change ( )A little better ( )Much better
2. Have the results of otoplasty made your overall life better or worse?
  ( )Much better ( )A little better ( )No change ( )A little worse ( )Much worse
3. Since your otoplasty, have you felt more or less optimistic about the future?
  ( ) Much more optimistic ( )More optimistic ( )No change ( )Less optimistic ( )Much less optimistic
4. Since your otoplasty, do you feel more or less embarrassed when with a group of people?
  ( )Much more embarrassed ( )More embarrassed ( )No change ( )Less embarrassed ( )Much less embarrassed
5. Since your otoplasty, do you have more or less self-confidence?
  ( )Much more self-confidence ( )More self-confidence ( )No change ( )Less self-confidence ( )Much less self-confidence
6. Since your otoplasty, have you found it easier or harder to deal with company?
  ( )Much easier ( )Easier ( )No change ( )Harder ( )Much harder
7. Since your otoplasty, do you feel you have more or less support from your friends?
  ( )Much more support More support ( )No change ( )Less support ( )Much less support
8. Have you been to your family doctor, for any reason, more or less often since your otoplasty?
  ( )Much more often ( )More often ( )No change ( )Less often ( )Much less often
9. Since your otoplasty, do you feel more or less confident about job opportunities?
  ( )Much more confident ( )More confident ( )No change ( )Less confident ( )Much less confident
10. Since your otoplasty, do you feel more or less self-conscious?
  ( )Much more self-conscious ( )More self/conscious ( )No change ( )Less self-conscious ( )Much less self-conscious
11. Since your otoplasty, are there more or fewer people who really care about you?
  ( )Many more people ( )More people ( )No change ( )Fewer people ( )Many fewer people
12. Since you had the otoplasty, do you catch colds or infections more or less often?
  ( )Much more often ( )More often ( )No change ( )Less often ( )Much less often
13. Have you taken more or less medicine for any reason, since your otoplasty?
  ( )Much more medicine ( )More medicine ( )No change ( )Less medicine ( )Much less medicine
14. Since your otoplasty, do you feel better or worse about yourself?
  ( )Much better ( )Better ( )No change ( )Worse ( )Much worse
15. Since your otoplasty, do you feel that you have had more or less support from your family?
  ( )Much more support ( )More support ( )No change ( )Less support ( )Much less support
16. Since your otoplasty, are you more or less inconvenienced by your protruding ears?
  ( )Much more inconvenienced ( )More inconvenienced ( )No change ( )Less inconvenienced ( )Much less inconvenienced
17. Since your otoplasty, have you been able to participate in more or fewer social activities?
  ( )Many more activities ( )More activities ( )No change ( )Fewer activities ( )Many fewer activities
18. Since your otoplasty, have you been more or less inclined to withdraw from social situations?
  ( )Much more inclined ( )More inclined ( )No change ( )Less inclined ( )Much less inclined
19. Are you satisfied with the result of your otoplasty?
  ( )Yes ( )No
20. Would you recommend our institution to other people (relatives and friends) to undergo the same procedure?
  ( )Yes ( )No
21. Would you undergo another esthetic procedure at our institution?
  ( )Yes ( )No
22. Did you find it hard to answer this questionnaire?
  ( )Yes ( )No
